# Prognostic value of the triglyceride—glucose index in non-muscle-invasive bladder cancer: a retrospective study

**DOI:** 10.3389/fnut.2024.1472104

**Published:** 2024-12-16

**Authors:** Yan Zhang, Xianfeng Shao, Li Ding, Wentao Xia, Kun Wang, Shan Jiang, Jiahao Wang, Junqi Wang

**Affiliations:** ^1^Department of Urology, The Affiliated Hospital of Xuzhou Medical University, Xuzhou, Jiangsu, China; ^2^Department of Urology, Shanghai Tenth People's Hospital, Shanghai, China

**Keywords:** bladder cancer, NMIBC, prognosis, TyG index, tumor recurrence, insulin resistance, nomogram

## Abstract

**Background:**

Bladder carcinoma is a type of urological tumor with high risks of recurrence and progression. The triglyceride-glucose (TyG) index has demonstrated significant promise as a prognostic marker for metabolic health in different types of cancer. Further research is needed to explore the relationships among non-muscle-invasive bladder cancer (NMIBC), the TyG index, and its prognostic importance. Purpose of this preliminary research is to assess the predictive significance of the TyG index for recurrence and progression risk in NMIBC patients.

**Methods:**

Data from patients admitted between October 2018 and July 2021 were reviewed, and there are 198 patients in total were included. The experimental data were supplied by medical records. In addition, patient prognoses were followed up via telephone. Furthermore, patients were separated into two groups: the high and low TyG groups, using X-tile software. Apart from recurrence-free survival (RFS), progression-free survival (PFS) was the main outcome. According to the TyG index, nomograms were also established.

**Results:**

The cohort consisted of 93 patients in the high TyG group and 105 patients in the low TyG group. The TyG index was a key prognostic factor for postoperative RFS (HR = 2.726, 95% CI = 1.474–5.041, *p* = 0.001) and PFS (HR = 2.846, 95% CI = 1.359–5.957, *p* = 0.006) among patients with NMIBC. The log-rank test revealed a notable disparity between the low and high TyG groups regarding RFS (*p* = 0.0025) and PFS (*p* = 0.0110). Moreover, it was strongly connected to well-known NMIBC risk factors. Because the TyG index exhibited good predictive value, the nomogram models were formulated.

**Conclusion:**

The TyG index serves as an isolated predictor of both RFS and PFS among patients with NMIBC, revealing new insights into disease treatment mechanisms. Indeed, the TyG index serves as a credible indicator of risk classification while facilitating early intervention among patients with NMIBC.

## 1 Introduction

Cancer of the bladder ranks ninth among the most common cancers worldwide (1). It predominantly manifests as urothelial carcinoma, which is divided into non-muscle invasive (NMIBC) and muscle-invasive (MIBC) categories on the basis of the depth of tumor invasion, which have distinctly different treatment approaches and prognoses (2). Initially, approximately 70–75% of patients are diagnosed with NMIBC (3). For bladder urothelial carcinoma, treatment approaches vary significantly depending on the depth of tumor infiltration. Despite the implementation of standard therapies such as transurethral resection of bladder tumors (TURBT) with bladder instillation therapy, tumor relapse or progression to the more aggressive MIBC occurs in 30–80% of patients (4). Therefore, identifying potential factors that impact patient prognosis post-TURBT is essential for improving treatment outcomes.

Insulin resistance (IR), a key part of metabolic syndrome (MetS) (5), refers to the diminished ability of an individual’s cells and tissues to respond effectively to insulin (6) and causes a compensatory rise in circulating insulin levels, thereby sustaining normoglycaemia (7). Increasing evidence indicates that IR is significantly related to a growing risk of developing some carcinomas and poor prognosis (5, 8, 9). MetS has been considered as a notable risk factor that adversely affects outcomes of bladder cancer patients (10–12). Components of MetS, such as diabetes mellitus and obesity, are significantly linked with the risk and prognosis of bladder carcinoma (13–17). Additionally, some studies have revealed that IR is more pervasive among bladder cancer patients than among those without (10). This evidence indicates that IR could be pivotal in bladder cancer progression, potentially significantly influencing patient prognosis. According to the established guidelines, the benchmark standard for identifying IR is the Euglycaemic-Hyperinsulinaemic Clamp (clamp-IR); nevertheless, the clamp-IR technique is seldom utilized in clinical practice because of its substantial cost and complexity (18). Compared with clamp-IR, the triglyceride-glucose (TyG) index may provide practical and dependable alternatives for evaluating IR during clinical sessions (19, 20).

Recent researches have underscored the TyG index as a potential prognostic marker for gastric, renal, and prostate cancer (21–23), indicating its potential role in early diagnosis and enhanced patient outcomes. Previous studies have investigated the disparity in the TyG index between populations with bladder carcinoma and those without. Tarantino et al. (24) found patients who have bladder carcinoma exhibited significantly higher serum TyG levels compared to those without the disease. However, the TyG index has not been explored in relation to its prognostic significance in NMIBC. It is hypothesized that an increased TyG index is believed to be strongly linked to a negative outcome in NMIBC patients.

This study aims at thoroughly investigating the prognostic relevance of the TyG index among NMIBC population, offering fresh insights into risk stratification and enhancing prognostic assessments for NMIBC patients, potentially informing more tailored treatment strategies and improving patient outcomes.

## 2 Materials and methods

### 2.1 Study design and population

A retrospective analysis was conducted at the Department of Urology, Affiliated Hospital of Xuzhou Medical University, involving surgical patients diagnosed with urothelial carcinoma of the bladder between October 2018 and the end of July 2020. The requirement for written informed consent has been waived due to the retrospective study design. This method ensured compliance with ethical standards while accommodating the study’s design limitations.

**Figure 1:**
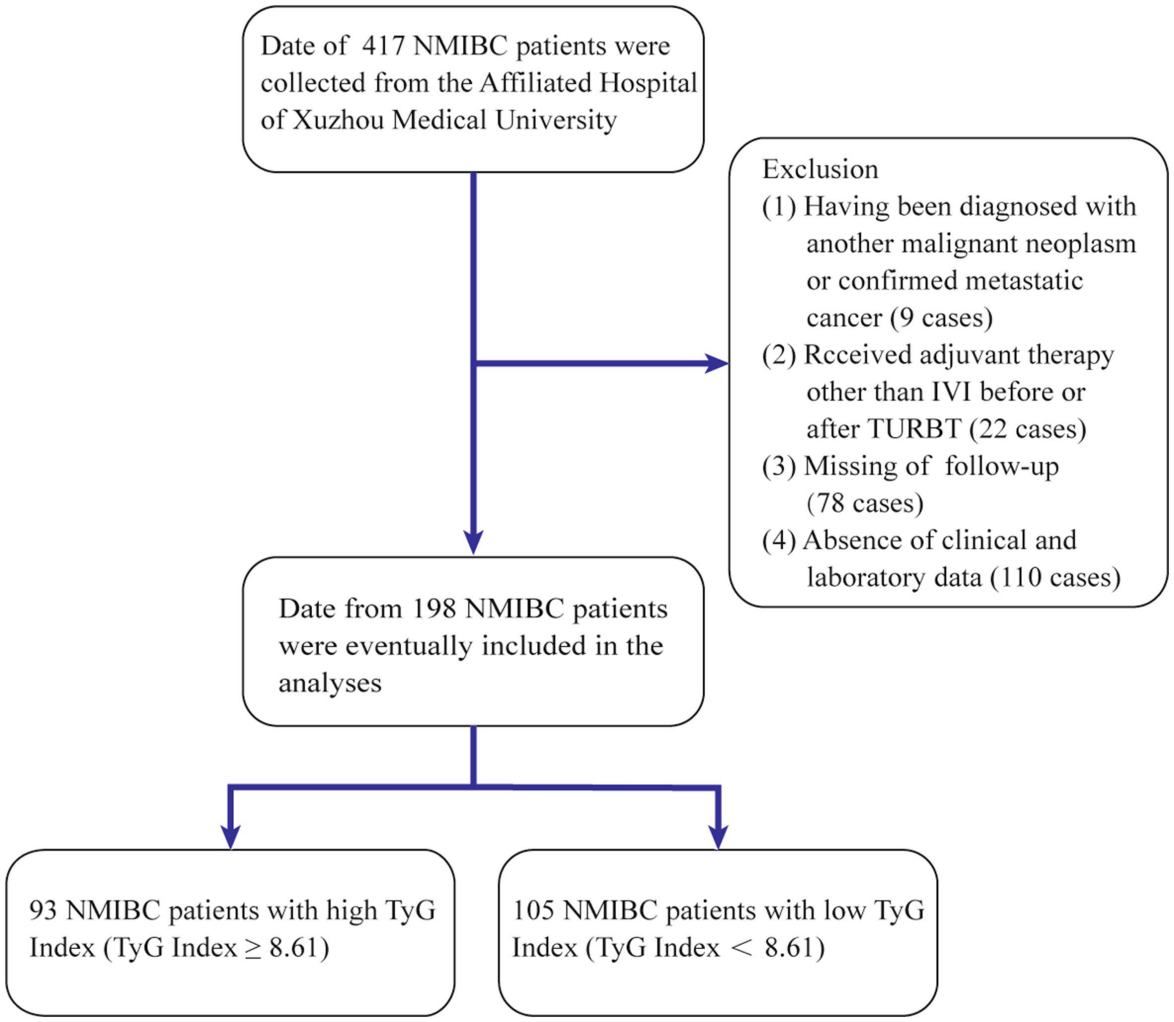
Process of clinical data collection and selection. NMIBC, non-muscle-invasive bladder cancer; IVI, intravesical instillation; TURBT, transurethral resection of the bladder tumor.

Data from patients diagnosed with bladder cancer between October 2018 and July 2020 were examined. Participants who satisfied the following criteria were enrolled: (1) underwent TURBT as a surgical procedure; (2) had pathological confirmation of urothelial carcinoma postsurgery; and (3) had specific pathological staging postsurgery. These patients were excluded: (1) with incomplete clinical information; (2) with other malignancies; and (3) who received adjuvant therapies but not bladder perfusion before or after TURBT. This study was approved by the Ethical Review Committee of the Affiliated Hospital of Xuzhou Medical University (approval number: XYFY2024-KL265-01), which conformed to the ethical guidelines of the 1975 Declaration of Helsinki. A retrospective evaluation was conducted on 417 patients with NMIBC within the specified period, and 198 patients were ultimately included (Figure 1).

### 2.2 Variables and definitions

In this study, various factors, including demographic elements such as age and sex, along with clinical parameters such as hypertension, diabetes, and smoking status, were analyzed. Metabolic indicators, such as triglycerides (TG) level, body mass index (BMI), high-density lipoprotein cholesterol (HDL-C) level, serum total cholesterol (TC) level, and the ratio of triglycerides to high-density lipoprotein cholesterol (TG/HDL-C), were also examined. Additional variables included hemoglobin (Hb) level, the TyG index, plasma albumin (Alb) level, and fasting plasma glucose (FPG) level. Tumor-specific characteristics, including the tumor size, pathology grade, T stage, tumor location, and number of tumors, were also assessed. All measurements and results were obtained from the most recent preoperative examination to ensure their accuracy and relevance to the patient’s current health status.

BMI = weight (kg)/height (m)^2^. Populations were categorized into two distinct groups: those with a BMI of 25 kg/m^2^ or higher were categorized as the high group, while those with a BMI of less than 25 kg/m^2^ were designated as the low group. Measurements were taken directly upon admission to ensure minimal bias.

TyG = ln [TG (mg/dL) × FBG (mg/dL)/2]. The preoperative TyG index critical point was determined using the enumeration method in the X-tile program^1^, selecting the value with the highest Youden index, which was identified as 8.61. Consequently, the individuals were classified into two distinct groups. The high TyG group included individuals with a TyG index of 8.61 or higher, indicating a greater risk of MetS and related complications. In contrast, the remaining individuals were placed in the low TyG group.

The TG/HDL-C ratio was divided into high (≥2.19) and low (<2.19) groups using the same method as the TyG index.

Pathologic staging was based on the Bladder Tumor, Lymph Node, and Metastasis Classification 2017, whereas histologic grading followed the World Health Organization 2004/2016 criteria.

### 2.3 Patient follow-up

In accordance with the follow-up guidelines for NMIBC, low-risk patients at our institution typically undergo their first post-operative evaluation 3 months after TURBT, followed by annual assessments. Intermediate- and high-risk patients, requiring more intensive monitoring, are evaluated every 3–6 months during the first 2 years, transitioning to annual check-ups thereafter. Diagnostic procedures, including blood and urine tests, are conducted alongside cystoscopy and urinary cytology to detect any potential recurrences. For patients at elevated risk of recurrence or disease progression, additional imaging through CT or MRI is utilized to exclude the presence of invasive disease. The time of TURBT was defined as the baseline for all patients. Following diagnosis, patients were monitored until the occurrence of high-grade recurrence, disease progression, or the conclusion of the follow-up period. For patients who experienced outcome events, such as recurrence or progression, the exit time was set as the date of the event. In contrast, for patients who did not experience any outcome events, the exit time was recorded as the date of their final follow-up. The clinical endpoints were recurrence-free survival (RFS) and progression-free survival (PFS). RFS was defined as the time from TURBT to the detection of high-grade recurrence, metastasis, or cancer-related death, while PFS referred to the time from surgery to clinical progression, metastasis, or cancer-related death.

### 2.4 Statistical analysis

R version 4.3.2 was utilized for all the statistical computations. To summarize the data, descriptive statistics were employed, including the calculation of the means, medians, interquartile ranges (IQRs), and proportions. Continuous variables were compared via either the Student’s *t*-test or the Mann–Whitney *U* test, depending on the distribution of the data, with the Mann–Whitney U test specifically applied to assess the differences in TyG index between BCG-unresponsive and BCG-responsive patients. The chi-square test or Fisher’s exact test was used to analyze categorical variables when necessary. Kaplan–Meier survival curves were created to calculate RFS and PFS, with group differences evaluated via the log-rank test. Univariate and multivariate Cox proportional hazards regression models were employed to discover autonomous prognostic determinants and present hazard ratios (HRs) along with 95% confidence intervals (95% CIs). Variables that had a *p-*value less than 0.05 during the univariate analysis were then incorporated into the multivariate analysis. Spearman’s rank correlation was used to analyze the relationships between the TyG index and various clinical and pathological factors, with a significance level of *p* < 0.05.

### 2.5 Development of the prognosis prediction models

Nomograms were developed to predict 1- to 3-year RFS and PFS via multivariate Cox regression analysis. These nomograms’ capacity to differentiate outcomes was assessed by determining the area under the receiver operating characteristic (ROC) curve (AUC), determining the models’ effectiveness in distinguishing different outcomes. Calibration curves were plotted to evaluate the concordance between the predicted and observed outcomes. To evaluate the alignment between the predicted and actual outcomes, calibration curves were generated. Internal validation of the nomograms was achieved via bootstrap resampling, which was repeated 1,000 times. To assess the practical application of the predictive models, decision curve analysis (DCA) was employed, which examined the net benefits across different threshold probabilities.

## 3 Results

### 3.1 Patient characteristics

This research involved 198 patients in total, all of whom underwent TURBT. The average age of individuals diagnosed with NMIBC was 67 years. These patients ranged in age from 56 to 76 years, with 168 (84.85%) males. Among them, 59 (29.80%) were hypertensive, and 20 (10.10%) were diabetic. The median BMI of the patients was 24.974 [22.491, 27.059] [IQR], and the median TyG index reached 8.550 [8.235, 8.925] [IQR]. The clinical and demographic characteristics of these two groups were then compared (Table 1). Key distinctions were discovered between the high and low TyG groups in several indices, including TC, TG, FPG, BMI, HDL-C, diabetes mellitus, TG/HDL-C proportion, Hb, Alb as a continuous variable, and hypertension (*p* < 0.05).

**Table 1:** Clinical characteristics and baseline demographics of NMIBC patients.

Variables	Overall (*n* = 198)	High TyG (*n* = 93)	Low TyG (*n* = 105)	*P*-value
Age (year)	67[58,76]	65[57,75]	68[63,78]	0.052
TC (mmol/l)	4.585[3.880,5.143]	4.780[4.150,5.515]	4.32[3.660,5.005]	<0.001
TG (mg/dl)	106.643[81.199,142.928]	147.79[126.998,188.505]	84.075[67.260.100.89]	<0.001
FBG (mg/dl)	93.060[84.420,104.085]	101.340[90.000,119.880]	88.380[82.440,96.200]	<0.001
HDL-C (mg/dl)	43.618[37.056,51.531]	40.916[33.775,46.899]	46.706[38.986,55.970]	<0.001
BMI	24.974[22.491,27.059]	25.690[23.223,27.494]	24.152[21.658,25.989]	0.001
TyG	8.550[8.235,8.925]	8.957[8.755,9.243]	8.252[7.976,8.405]	<0.001
TG/HDL-C	2.391[1.696,3.780]	3.701[2.758,5.214]	1.728[1.285,2.237]	<0.001
Hb (g/l)	139.000[129.000,149.000]	144[132,152.5]	136[124,145]	<0.001
Alb (g/l)	43.400[40.900,45.700]	43.500[41.750,47.000]	42.800[40.250,45.250]	0.013
Maximum tumor diameter (mm)	19[13.75,27.25]	18[14,28]	20[13,27.5]	0.442
Age (year), *n* (%)				0.259
<70	113(57.071)	57(61.290)	56(53.333)	
≥70	85(42.929)	36(38.710)	49(46.667)	
BMI, *n* (%)				<0.001
<25	102(51.515)	36(38.710)	66(62.857)	
≥25	96(48.485)	57(61.290)	39(37.143)	
Hb (g/l), *n* (%)				0.041
<136	81(40.909)	31(33.333)	50(47.619)	
≥136	117(59.091)	62(66.667)	55(52.381)	
Alb (g/l), n (%)				0.194
<43.4	97(48.990)	41(44.086)	56(53.333)	
≥43.4	101(51.010)	52(55.914)	49(46.667)	
Gender, *n* (%)				0.971
Male	168(84.848)	79(84.946)	89(84.762)	
Female	30(15.152)	14(15.054)	16(15.238)	
Hypertension, *n* (%)				0.023
No	139(70.202)	58(62.366)	81(77.143)	
Yes	59(29.798)	35(37.634)	24(22.857)	
Diabetes, *n* (%)				0.030
No	178(89.900)	79(84.946)	99(94.286)	
Yes	20(10.100)	14(15.054)	6(5.714)	
Smoking status, *n* (%)				0.790
No	113(57.070)	54(58.065)	59(56.190)	
Yes	85(42.930)	39(41.935)	46(43.810)	
Bladder neck invasion, *n* (%)				0.339
No	163(82.323)	74(79.570)	89(84.762)	
Yes	35(17.677)	19(20.430)	16(15.238)	
Tumor number, *n* (%)				0.265
Single	96(48.484)	49(52.688)	47(44.762)	
Multiple	102(51.516)	44(47.312)	58(55.238)	
Pathology grade, *n* (%)				0.546
Low-grade	66(33.333)	33(35.484)	33(31.429)	
High-grade	132(66.667)	60(64.516)	72(68.571)	
T category, *n* (%)				0.656
Ta	114(57.576)	52(55.914)	62(59.048)	
T1	84(42.424)	41(44.086)	43(40.952)	
Prior recurrence status, *n* (%)				0.227
Primary	176(88.889)	80(86.022)	96(91.429)	
Recurrent	22(11.111)	13(13.978)	9(8.571)	

Median [IQR] is used to present continuous variables, while percentages are used to present categorical variables.

TG, triglyceride; TC, serum total cholesterol; HDL-C, high-density lipoprotein cholesterol; BMI, body mass index; Hb, hemoglobin; Alb, albumin; FBG, fasting blood glucose.

### 3.2 TyG index and patient prognosis

**Figure 2:**
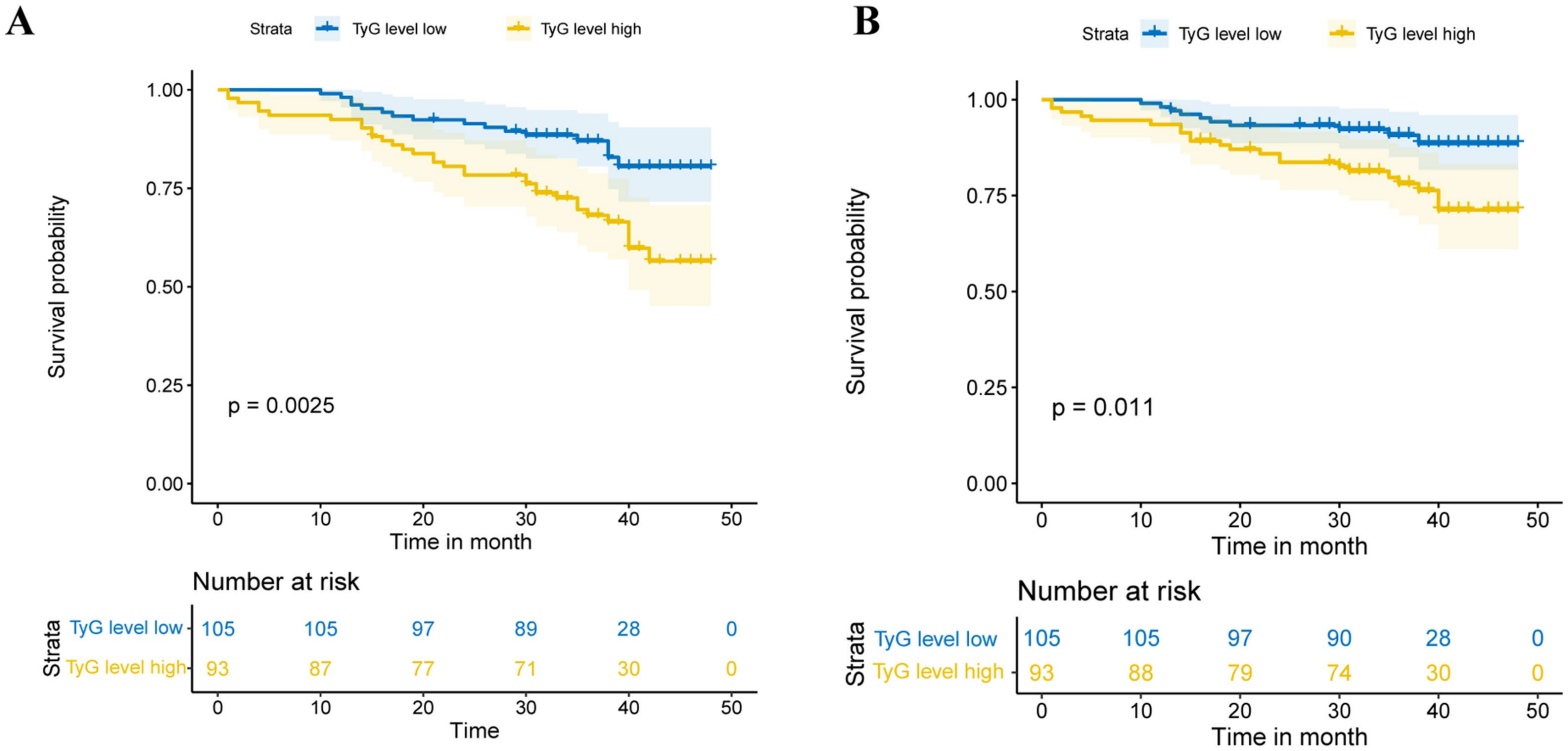
Kaplan–Meier curves in terms of RFS **(A)** and PFS **(B)**.

Patients had varying lengths of follow-up periods, ranging from 1 to 48 months, with a median follow-up time of 36 months. The average follow-up duration was 35 months, providing a comprehensive assessment of patient outcomes over a significant period. During this period, 49 cases (24.74%) of tumor recurrence and 34 cases (17.17%) of tumor progression were observed. RFS and PFS were evaluated through Kaplan–Meier survival curves (Figure 2), which revealed that the TyG index is negatively correlated with patient prognosis.

The variables influencing patient prognosis were meticulously analyzed via univariate and multivariate Cox regression models. This in-depth exploration aimed to uncover their associations with RFS and PFS, thereby providing a robust understanding of the factors impacting patient outcomes. In the univariate Cox regression analysis (Tables 2, 3), seven factors were strongly associated with RFS: the TyG index, maximum diameter of the tumor, prior recurrence status, bladder neck invasion, tumor number, pathological grade, and T stage. The results demonstrated a relationship between the TyG index and recurrence within NMIBC populations, indicating its possible use as a predictive indicator for recurrence in such instances (HR = 2.432, 95% CI = 1.338–4.422, *p* = 0.004). Similarly, when PFS was used as the outcome, the same factors influenced NMIBC progression. If the TyG index was more elevated, the progression easily happened (HR = 2.402, 95% CI = 1.170–4.930, *p* = 0.017).

**Table 2:** Univariable and multivariable analysis for predicting recurrence-free survival.

Variables	Univariate RFS HR (95% CI)	*P*-value	Multivariate RFS HR (95% CI)	*P*-value
Age (year)		0.506		
<70	1 (reference)			
≥70	1.210 (0.689–2.124)			
Gender		0.490		
Male	1 (reference)			
Female	1.351 (0.575–3.174)			
BMI		0.766		
<25	1 (reference)			
≥25	1.089 (0.622–1.907)			
TyG		0.004		0.001
<8.61	1 (reference)		1 (reference)	
≥8.61	2.432 (1.338–4.422)		2.726 (1.474–5.041)	
Hb (g/l)		0.370		
<136	1 (reference)			
≥136	0.774 (0.441–1.356)			
Alb (g/l)		0.737		
<43.4	1 (reference)			
≥43.4	1.102 (0.627–1.937)			
Maximum tumor diameter (mm)	1.025 (1.003–1.046)	0.023	1.021 (0.997–1.045)	0.084
Prior recurrence status		<0.001		<0.001
Primary	1 (reference)		1 (reference)	
Recurrent	4.976 (2.693–9.195)		4.319 (2.295–8.130)	
Bladder neck invasion		0.003		0.580
No	1 (reference)		1 (reference)	
Yes	2.438 (1.341–4.430)		1.205 (0.622–2.333)	
Hypertension		0.520		
No	1 (reference)			
Yes	1.217 (0.669–2.212)			
Diabetes		0.740		
No	1 (reference)			
Yes	0.855 (0.338–2.159)			
Smoking status		0.595		
No	1 (reference)			
Yes	1.165 (0.663–2.047)			
Tumor number		0.012		0.035
Single	1 (reference)		1 (reference)	
Multiple	2.174 (1.183–3.994)		2.114 (1.056–4.232)	
Pathology grade		0.021		0.333
Low-grade	1 (reference)		1 (reference)	
High-grade	2.348 (1.139–4.840)		1.502 (0.660–3.418)	
T category		0.010		0.261
Ta	1 (reference)		1 (reference)	
T1	2.107 (1.191–3.728)		1.442 (0.762–2.728)	

BMI, body mass index; Hb, hemoglobin; Alb, albumin.

**Table 3:** Univariable and multivariable analysis for predicting progression-free survival.

Variables	Univariate RFS HR (95% CI)	*P*-value	Multivariate RFS HR (95% CI)	*P*-value
Age (year)		0.366		
<70	1 (reference)			
≥70	1.365(0.696–2.678)			
Gender		0.741		
Male	1 (reference)			
Female	0.862(0.357–2.081)			
BMI		0.807		
<25	1 (reference)			
≥25	0.92(0.469–1.803)			
TyG		0.017		0.006
<8.61	1 (reference)		1 (reference)	
≥8.61	2.402(1.170–4.930)		2.846(1.359–5.957)	
Hb (g/l)		0.064		
<136	1 (reference)			
≥136	0.527(0.268–1.038)			
Alb (g/l)		0.752		
<43.4	1 (reference)			
≥43.4	0.897(0.457–1.760)			
Maximum tumor diameter (mm)	1.031(1.007–1.056)	0.012	1.032(1.003–1.062)	0.028
Prior recurrence status		0.001		0.008
Primary	1 (reference)		1 (reference)	
Recurrent	3.532(1.643–7.590)		2.928(1.327–6.462)	
Bladder neck invasion		0.005		0.718
No	1 (reference)		1 (reference)	
Yes	2.76(1.365–5.579)		1.154(0.529–2.518)	
Hypertension		0.913		
No	1 (reference)			
Yes	1.0042(0.498–2.180)			
Diabetes		0.987		
No	1 (reference)			
Yes	1.009(0.355–2.867)			
Smoking status		0.813		
No	1 (reference)			
Yes	1.085(0.5510–2.136)			
Tumor number		0.004		0.008
Single	1 (reference)		1 (reference)	
Multiple	3.167(1.433–6.998)		3.338(1.370–8.134)	
Pathology grade		0.02		0.281
Low-grade	1 (reference)		1 (reference)	
High-grade	3.095(1.198–7.996)		1.783(0.623–5.106)	
T category		0.017		0.474
Ta	1 (reference)		1 (reference)	
T1	2.331(1.167–4.658)		1.324(0.614–2.854)	

BMI, body mass index; Hb, hemoglobin; Alb, albumin.

**Figure 3:**
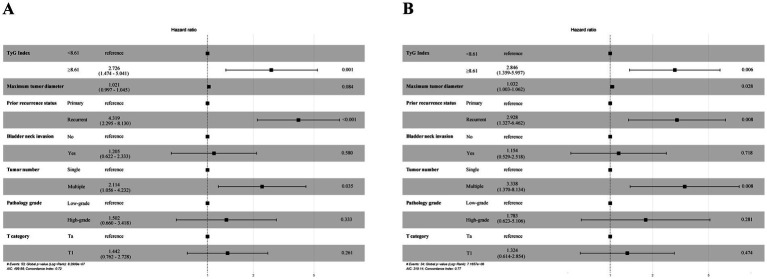
Multivariate Cox proportional hazards regression analyses according to RFS **(A)** and PFS **(B)**.

In the analyses via multivariate Cox regression (Tables 2, 3), the TyG index, maximum tumor diameter, prior recurrence status, bladder neck invasion, pathological grade, tumor number, and T stage were included. Accordingly, the TyG index (HR = 2.726, 95% CI = 1.474–5.041, *p* = 0.001), prior recurrence (HR = 4.319, 95% CI = 2.295–8.130, *p* < 0.001), and tumor number (HR = 2.114, 95% CI = 1.056–4.232, *p* = 0.035) were key independent risk factors for NMIBC recurrence (Figure 3). When disease progression was used as an outcome variable, the TyG index (HR = 2.846, 95% CI = 1.359–5.957, *p* = 0.006), maximum tumor diameter (HR = 1.032, 95% CI = 1.003–1.062, *p* = 0.028), prior recurrence status (HR = 2.928, 95% CI = 1.327–6.462, *p* = 0.008), and tumor number (HR = 3.338, 95% CI = 1.370–8.134, *p* = 0.008) were considered isolated risk factors for bladder cancer progression (Figure 3).

### 3.3 Relationship between TyG index and bacillus Calmette-Guérin (BCG) unresponsiveness in NMIBC

The BCG intravesical therapy plays a vital role in the management of high-grade NMIBC. Despite its importance, only 32 out of 132 patients received this treatment, primarily due to patient-specific economic factors. Among those who underwent BCG therapy, 13 patients experienced a recurrence of bladder cancer during treatment, yielding a recurrence rate of 40.62%. Comparative analysis between the 13 BCG-unresponsive patients and the 19 patients who did not experience recurrence revealed that the TyG index was significantly higher in the BCG-unresponsive group (*p* = 0.025) (Supplementary Figure S1).

### 3.4 Assessment of repeat TURBT in pT1 patients

The EAU guidelines recommend that NMIBC patients with confirmed T1 stage disease following an initial TURBT undergo a repeat TURBT within 2–6 weeks to enhance diagnostic accuracy and ensure comprehensive staging. In this study’s cohort of 84 T1 stage patients, 28 underwent a second TURBT, with complete tumor resection and muscle sampling confirmed during the initial procedure. When analyzed using RFS and PFS as primary endpoints, the TyG index did not emerge as an independent prognostic factor, nor did repeat TURBT show a significant association with improved RFS (HR = 0.425, 95% CI = 0.173–1.047, *p* = 0.063) (Supplementary Table S1) or PFS (HR = 0.405, 95% CI = 0.136–1.206, *p* = 0.104) (Supplementary Table S2).

### 3.5 Subgroup analysis and interactions

**Figure 4:**
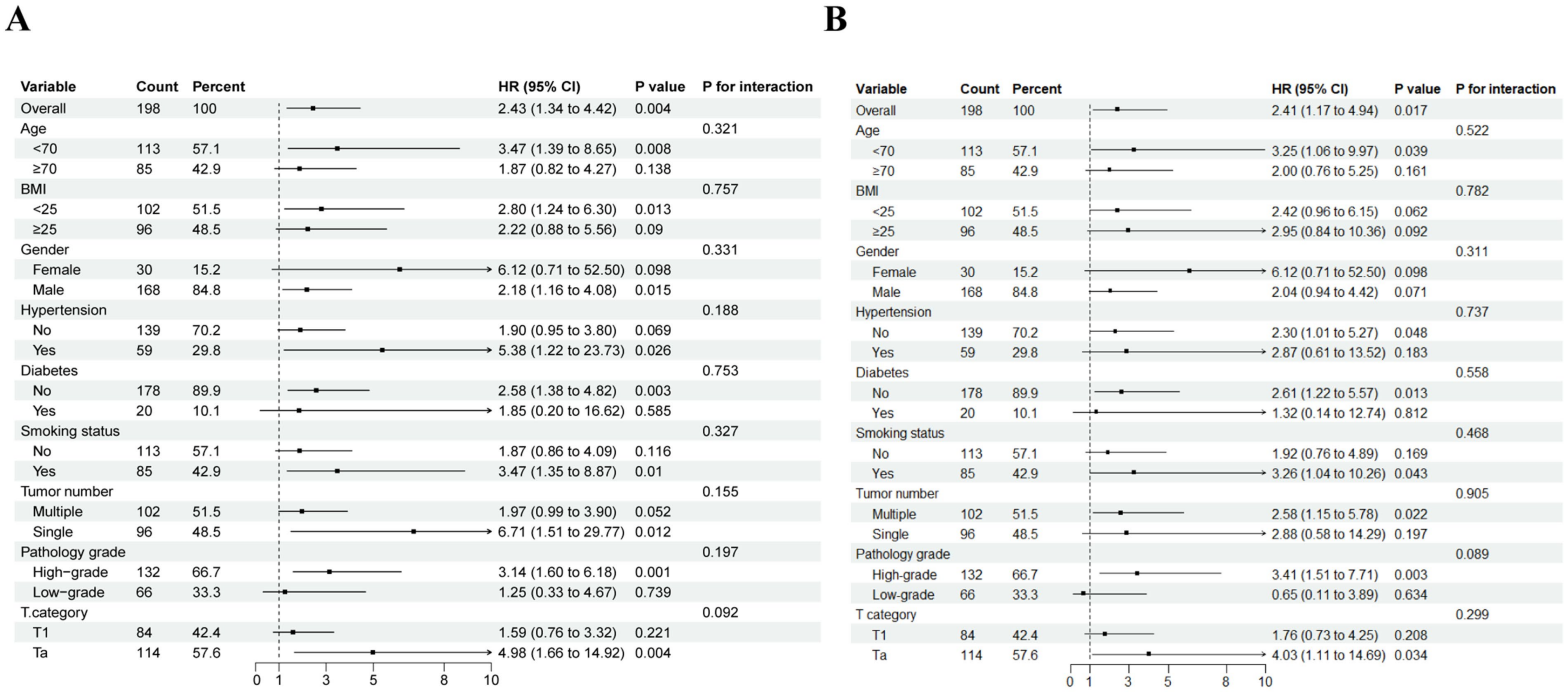
Subgroup analysis and interaction analysis based on RFS **(A)** and PFS **(B)**. BMI, body mass index.

An additional investigation was conducted into the correlation between NMIBC prognosis and the TyG index through subgroup analysis (Figure 4). This analysis revealed that RFS reached statistical significance among the following subgroups: males, age < 70 years, BMI < 25 kg/m^2^, history of previous smoking, no history of diabetes, single tumor, high-grade pathology, and Ta stage. Similarly, PFS was found to be statistically significant in the subgroups with no history of hypertension, no history of diabetes, multiple tumors, high pathological grade, and Ta stage. No notable interaction emerged between the two subgroups (total *p-*values >0.05).

### 3.6 Correlation study among the TyG index and prognostic elements for NMIBC

This analysis enabled us to evaluate both the magnitude and direction of the relationships involving the TyG index and various biochemical parameters, providing a comprehensive understanding of its potential. As a result, prominent correlations were proven linking the TyG index with Hb, HDL-C, BMI, Alb, as well as TC (*p* < 0.05). There were no important correlations among the other factors (Table 4).

**Table 4:** Correlations between the TyG index and NMIBC risk factors.

Elements	Correlation coefficient (*r*)	*P*-value
BMI	0.336	**<0.001**
Hb	0.279	**<0.001**
Alb	0.176	**0.013**
HDL-C	−0.343	**<0.001**
TC	0.295	**<0.001**
TG/HDL-C	0.819	**<0.001**
Maximum tumor diameter (mm)	−0.059	0.408
Tumor number	HR = 0.679 (0.404–1.143)	0.145
Pathology grade	HR = 1.071 (0.622–1.845)	0.804
T category	HR = 1.219 (0.727–2.044)	0.453
Prior recurrence status	HR = 1.242 (0.561–2.751)	0.593
Bladder neck invasion	HR = 1.444 (0.751–2.778)	0.271

TC, serum total cholesterol; HDL-C, high-density lipoprotein cholesterol; BMI, body mass index; Hb, hemoglobin; Alb, albumin. Bold values mean the difference is statistically significant (*p* < 0.05).

### 3.7 Building the nomograms

**Figure 5:**
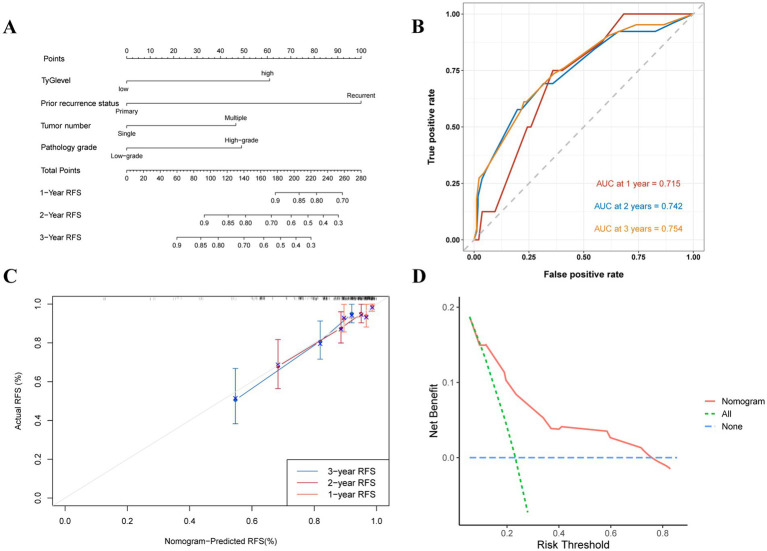
Evaluation of the TyG index for predicting 1 to 3-year RFS in NMIBC patients. **(A)** Nomogram for predicting individual recurrence risk, with each variable contributing to the overall risk score. **(B)** ROC curve showing the performance of the TyG index in predicting PFS, with AUC values of 0.715, 0.742, and 0.754 for the 1- to 3-year RFS rates, indicating good discrimination ability. **(C)** Calibration curve assessing the agreement between predicted and observed recurrence rates. **(D)** DCA demonstrating the net clinical benefit of using the TyG index at various threshold probabilities, with the model providing higher net benefit compared to the “treat all” and “treat none” strategies.

**Figure 6:**
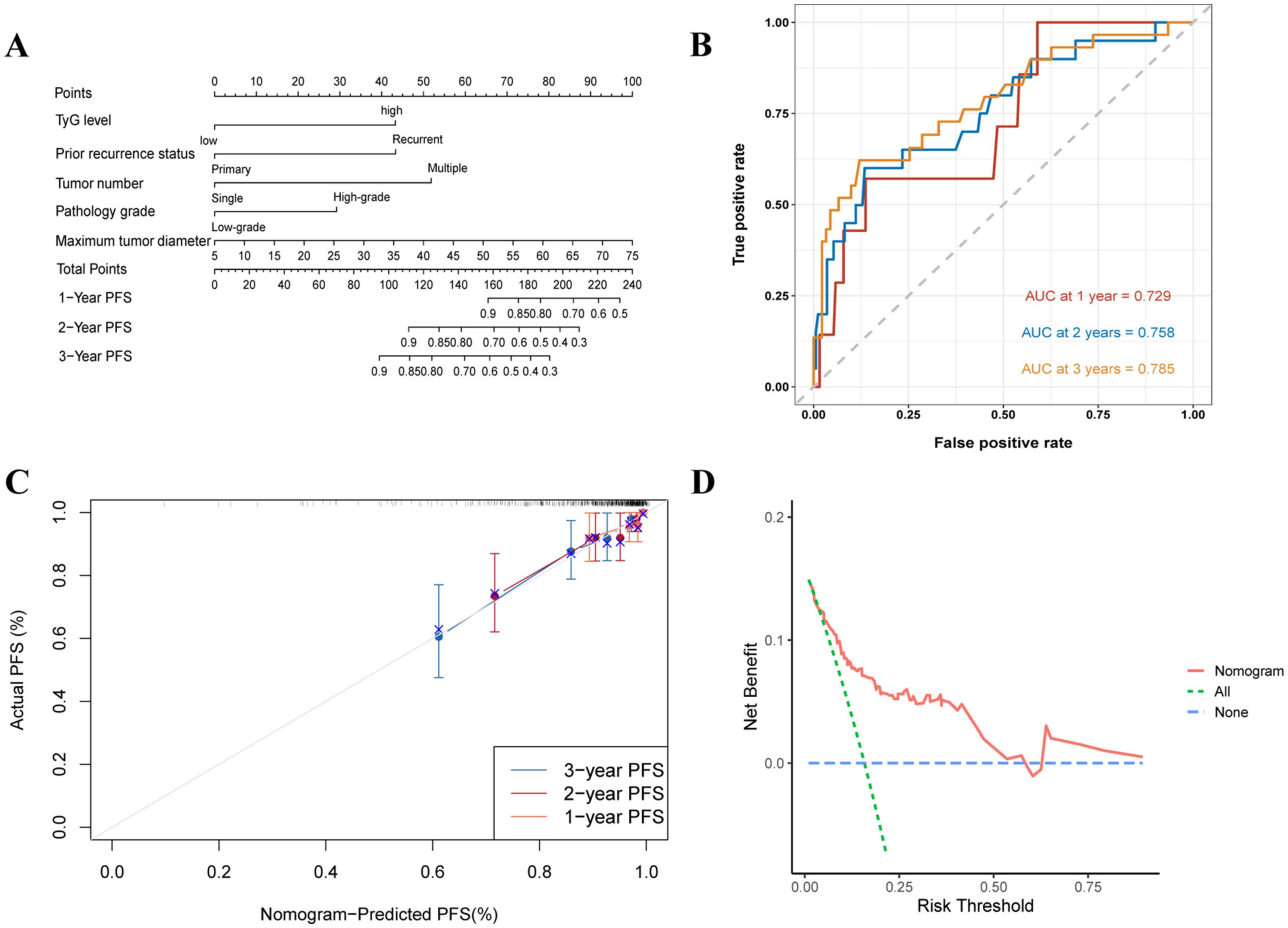
Evaluation of the TyG index for predicting 1 to 3-year PFS in NMIBC patients. **(A)** Nomogram for predicting individual progression risk, with each variable contributing to the overall risk score. **(B)** ROC curve showing the performance of the TyG index in predicting PFS, with AUC values of 0.729, 0.758, and 0.785 for the 1- to 3-year PFS rates, indicating good discrimination ability. **(C)** Calibration curve assessing the agreement between predicted and observed progression rates. **(D)** DCA demonstrating the net clinical benefit of using the TyG index at various threshold probabilities, with the model providing higher net benefit compared to the “treat all” and “treat none” strategies.

Nomograms were built to predict 1- to 3-year RFS and PFS rates according to the multivariate Cox regression and factors previously shown, with the aim of influencing NMIBC patient prognosis through the TyG index (Figures 5A, 6A). The ROC curves showed strong predictive accuracy for the model, with AUC values of 0.729, 0.758, and 0.785 for the 1- to 3-year PFS rates (Figure 6B). For the 1- to 3-year RFS predictions, the values were 0.715, 0.742, and 0.754 (Figure 5B), indicating strong predictive performance for both models. Verified by 1,000 bootstrap resamples, the model reliability was confirmed via calibration (Figures 5C, 6C). Decision curve analysis (DCA) revealed that patients could derive significant clinical benefit from the models (Figures 5D, 6D).

## 4 Discussion

The study is the pioneer in investigating the impact of the TyG index on the outcomes of populations with NMIBC, yielding findings that are clinically important. Elevated TyG index values were correlated with a poorer prognosis among patients with NMIBC, indicating that it is an isolated risk factor for forecasting not only postoperative relapse but also development. This study identified critical threshold values for the TyG index, enabling the stratification of NMIBC patients into high- and low-risk categories for recurrence and progression. Significant associations were observed between the TyG index and NMIBC prognosis, particularly in subgroups such as males, patients under 70, smokers, non-diabetic individuals, those with single tumors, high-grade pathology, and those with a BMI < 25 kg/m^2^. Additionally, the TyG index showed significant correlations with several clinical factors known to influence bladder cancer prognosis, underscoring its prognostic value in NMIBC patients.

Previous studies have shown a strong association between MetS and the progression of several cancers, including prostate, hepatocellular, renal cell, bladder, and gastric carcinoma (5, 8, 9, 25). IR, a key component of metabolic syndrome (15), is a key player in the progression of malignant tumors, such as bladder carcinoma (26, 27). To diagnose IR, the clinical gold standard has become clamp-IR. Nonetheless, the more commonly used clamp-IR is costly, challenging to perform, and susceptible to interference from insulin injections (10, 18). The TyG index, a newly introduced and easily measurable index, has shown superior predictive value (19, 20). Owing to its simplicity and effectiveness, the TyG index has replaced traditional indices of IR and is regarded as an important risk factor for several tumors. For example, as recognized by Kim et al. (21), there is a crucial relationship between gastric cancer and high TyG index levels. Similarly, the TyG index has been found that it can independently predict the outcomes in renal cell carcinoma patients after surgery (22). However, there is still a research gap in the TyG index along with the endpoints of bladder carcinoma patients. This presence of this gap underscores the need for a comprehensive study aimed at elucidating the prognostic significance of the TyG index among NMIBC populations.

This index is correlated with several metabolic factors, such as HDL-C, TC, BMI, Hb, and Alb, along with hypertension, all of which are recognized prognostic factors for bladder cancer (17, 26–29). Thus, the TyG index can act as an effective supplementary tool for predicting NMIBC prognosis. Survival analysis revealed significantly higher recurrence and disease progression rates in the high TyG group, highlighting its strong prognostic significance. The TyG index acts as an agent biomarker and is strongly related to MetS, suggesting that NMIBC patients with metabolic syndrome may have a worse prognosis, which aligns with the findings of previous studies (13, 30). Garg et al. (31) demonstrated that traditional indicators of metabolic syndrome, including hyperlipidaemia, hypertension, and diabetes mellitus, or a BMI ≥30 kg/m^2^, are not reliable predictors of relapse among elderly NMIBC patients. In this cohort of 198 patients, BMI, hypertension, and diabetes mellitus were not regarded as independent risk factors for NMIBC prognosis, but the TyG index could be, underscoring its prognostic value. Accordingly, this index is a more precise prognostic index among NMIBC patients, offering higher specificity than traditional MetS markers, thus serving as a valuable tool for clinical risk stratification.

Intravesical BCG therapy is the standard treatment for high-grade NMIBC. Nonetheless, 30–40% of patients may still experience recurrence despite receiving adequate treatment. The EAU guidelines recommend radical cystectomy (RC) for patients with BCG-unresponsive disease; however, this surgical intervention can significantly impact quality of life. Recent studies have also identified several potential therapeutic targets for BCG-unresponsive NMIBC (32, 33). A key focus of our current research is how to early identify this specific patient population. This study demonstrates that BCG-unresponsive NMIBC patients exhibit a higher TyG index compared to those without recurrence. BCG therapy functions by promoting Th1 cell polarization and activating local immune responses to inhibit the tumor microenvironment (34). In contrast, patients with MetS often exhibit leukocyte activation and functional impairments, which diminish their ability to suppress tumors (35). The TyG index serves as a numerical reflection of this condition. Consequently, a higher TyG index may indicate an increased risk of recurrence, particularly among BCG-unresponsive patients. This underscores the potential of the TyG index as a valuable tool for predicting patient responses to BCG therapy.

In this cohort of T1 NMIBC patients, the TyG index did not serve as a dependable prognostic marker. Additionally, a repeat TURBT was not associated with enhanced survival outcomes for this patient subset. Consistent with our results, a large multicenter retrospective study led by Beijert et al. (36) similarly concluded that repeat TURBT in T1 NMIBC patients, after initial complete resection with confirmed muscle presence, did not yield a significant prognostic advantage. Despite the EAU guidelines recommending a repeat TURBT within 2–6 weeks postoperatively, concerns remain regarding its potential to delay critical adjuvant treatments, such as BCG instillation, which may complicate its role in improving long-term prognosis. Thus, determining the advisability of a repeat TURBT in T1 patients warrants a comprehensive assessment of its potential advantages and inherent risks. Apart from this, in the analysis utilizing RFS and PFS as endpoints, this study found that the 2004/2016 bladder cancer grading system has limited effectiveness in predicting clinical outcomes for NMIBC patients, challenging common assumptions. In contrast, research led by the EAU group indicates that the 1973 grading system is more effective at predicting progression in NMIBC patients, particularly within small high-risk populations (37). The prognostic analysis results for the 2004/2016 grading system were not significant, further validating this assertion. Therefore, when evaluating NMIBC prognosis, it is essential to consider various influencing factors, and the TyG index may provide important supplementary information.

Obesity, a significant component of MetS, is generally linked to a poorer postoperative prognosis in bladder cancer patients. Nevertheless, the subgroup examination revealed that the TyG indicator was notably linked to the likelihood of NMIBC relapse solely in populations whose BMI was below 25 kg/m^2^. This difference does not seem obvious among individuals with a high BMI. This finding adds to the growing evidence supporting the “obesity paradox,” suggesting that NMIBC patients with obesity may experience a better prognosis (38, 39). Similar conclusions have been drawn in other studies, further supporting our finding (17, 40, 41). The mechanisms by which obesity might reduce recurrence risk in NMIBC patients are not fully understood. One potential protective mechanism involves elevated levels of proinflammatory molecules produced by adipose tissue, including lipocalins, cytokines, and leptin (42). For example, leptin has demonstrated antitumor effects by improving the activation and growth of natural killer cells (43). The obesity paradox may also be caused by the physiological adaptations to insulin resistance observed in obese individuals (22). Additionally, a higher BMI often reflects better nutritional status, which can influence immune status and local tumor immunity, thereby affecting prognosis (44). However, other researchers argue that BMI alone is an inadequate measure of obesity, as various confounding factors—including social and economic status, exercise or bodily activity, and eating habits—may influence outcomes (45). Therefore, it remains premature to conclude that obesity directly improves BCa prognosis (46). While the “obesity paradox” remains a topic of debate, this study indicates that abnormal IR in patients without obesity is more strongly linked to NMIBC prognosis, underscoring the importance of heightened clinical vigilance in this subgroup. Sex differences in cancer risk related to IR have also been documented. As discovered by Häggström et al. (27), MetS was highly connected to an intense hazard of urothelial carcinoma in males but not females in a study involving 580,000 individuals. This may be correlated with higher testosterone levels and greater bioavailability among males, which stimulates epithelial cell proliferation (47, 48), potentially explaining the sex differences observed in our subgroup analyses. Furthermore, follow-up revealed that patients with hypertension and diabetes actively controlled their blood glucose and blood pressure through diet and medication. In contrast, non-diabetic and non-hypertensive patients did not actively monitor their status. The TyG index has been shown to be more efficient in sensing MetS among non-diabetic populations (20).

Examining the correlation between IR and bladder carcinoma can expand our comprehension of the biological mechanisms through which the TyG index can predict NMIBC prognosis. Recent studies have focused mainly on controlling hyperinsulinaemia and exploring the insulin-like growth factor-1 (IGF-1) related to tumor promotion caused by IR. First, insulin is capable of binding to and activating the IGF-1 receptor, which acts as a growth factor to increase mitosis through the induction of downstream signaling pathways (49). Second, insulin resistance causes high insulin levels in the blood. Hyperinsulinaemia promotes tumorigenesis and progression through the suppression of compounds encoding IGF-binding proteins, thereby increasing IGF activity (50). Moreover, high blood sugar levels can hinder the function of mitochondria, causing a decrease in DNA repair and promoting reactive oxygen species (ROS) generation, leading to cellular damage (51). Thus, the relationship between IR and tumors is complex, with these pathways synergistically promoting tumorigenesis (52, 53).

Research has shown that metformin inhibits bladder cancer development by targeting the PI3k-Akt–mTOR signaling pathway (17, 54). Ferro et al. (55) also reported that statins significantly decrease the risk of relapse following elective NMIBC resection. Furthermore, recent studies have indicated that prolonged high levels of blood glucose and lipids are linked to poor prognosis in patients with bladder cancer (17, 25, 26). Current research does not directly link postoperative changes in the TyG index to prognosis in NMIBC patients. However, our findings suggest a potential association between the preoperative TyG index and patient outcomes. This underscores the importance of monitoring blood glucose and lipid levels, as lowering the TyG index may improve survival. Future studies should explore how changes in the TyG index after surgery affect the prognosis of NMIBC patients.

The 2021 EAU guidelines established a very high-risk (VHR) group for NMIBC, which includes high-grade T1 carcinoma *in situ* (CIS) patients, individuals with lymphovascular invasion (LVI), those with specific urothelial carcinoma subtypes, and patients with prostatic urethral CIS (56). However, most VHR NMIBC patients lack preoperative metrics for MetS, such as blood lipid levels, leading to their exclusion from our study. The presence of variant histological subtypes is recognized as a critical factor influencing NMIBC prognosis. According to the WHO 2022 classification, urothelial carcinoma subtypes comprise atypical tumors and micropapillary carcinoma. These subtypes can exhibit significant variations in biological behavior, clinical characteristics, and treatment responses (57). Research by Lopez-Beltran et al. (58) highlights those patients with micropapillary, nested, or basaloid, as well as glandular differentiation, tend to have poorer prognoses, particularly in the case of micropapillary carcinoma, which shows limited responsiveness to BCG therapy. This underscores the importance of considering these subtypes in the development of personalized treatment strategies in clinical practice. LVI is another crucial prognostic factor for NMIBC and serves as a primary indicator of muscle invasion after TURBT. Furthermore, LVI may assist in guiding treatment decisions for urothelial carcinoma patients by identifying those who could avoid unnecessary adjuvant chemotherapy (59). However, due to the complexities involved in evaluating LVI in TURBT specimens and the lack of standardized assessment criteria, our center does not routinely report LVI in post-TURBT pathology reports. As a result, LVI may be present among the included patients, which represents a limitation of this study. Prostatic urethral CIS, while relatively uncommon, poses significant diagnostic challenges. According to Palou et al. (60), prostatic urethral CIS is the sole prognostic factor affecting the outcomes of T1 high-grade bladder tumors treated with BCG, indicating that its presence correlates with a poorer prognosis. Typically, RC is the primary treatment for VHR NMIBC patients. However, findings by Contieri et al. (56) suggest that intravesical BCG therapy can yield prognostic results comparable to those of RC in these patients. Consequently, intravesical BCG could serve as a viable and safe alternative to early RC, offering an initial treatment option for VHR NMIBC patients. As we expand our sample size, we are collaborating with pathologists to reevaluate these specimens. In future studies with larger cohorts, the TyG index will be employed to enhance risk stratification for these patients.

The findings of this research hold significant implications for clinical practice. The TyG index has been identified as a potential biomarker that can help in recognizing individuals at high risk of recurrence and progression post-TURBT, enabling proactive preventive measures. Utilizing the nomograms, clinicians can preoperatively identify high-risk bladder cancer patients with poor prognosis and implement more rigorous postoperative follow-up for timely intervention. For patients who are unable to effectively manage their diet preoperatively, medications such as metformin and statins can be prescribed to regulate blood glucose and lipid levels, thereby offering survival benefits. These measures are especially crucial for patients with a low BMI, as they may be less likely to monitor their blood glucose and lipid levels compared to obese patients.

As a real-world study, each patient was rigorously followed up with regular preoperative tests, treatments, and prognostic evaluations, thereby enhancing the reliability of the findings. However, this study has several limitations. First, being retrospective and conducted at a single center, it may introduce bias due to the limited geographic and demographic diversity of the patient cohort. Secondly, the cut-off point determined using X-tile intercepts was based solely on data from our institution, potentially limiting the generalizability of the findings. Additionally, the wide range of follow-up times (1–48 months) may have introduced bias, as patients with shorter follow-up durations had less opportunity for recurrence or progression, thereby affecting the comparability of outcomes. Furthermore, the study did not examine dynamic variations in the TyG index and their potential impact on RFS and PFS. Moreover, it did not include the subset of NMIBC patients classified as very high risk by the EAU, which leaves the role of the TyG index in stratifying these patients unclear. Future studies should prioritize large-sample, multicenter prospective cohorts which contain various risk stratifications and include an analysis of TyG index variations over time, which may help define an optimal TyG index range for predicting prognosis in NMIBC patients.

## 5 Conclusion

This research revealed that the TyG index is an important predictor of both progression and recurrence among NMIBC patients. Clinically, people with high TyG levels face a greater chance of experiencing postsurgery recurrence and disease advancement. Nomograms with basis of the TyG index facilitate early detection of high-risk population with unfavorable prognoses, thereby allowing for more rigorous monitoring and proactive intervention strategies, ultimately enhancing patient survival outcomes.

## Data Availability

The data analyzed in this study is subject to the following licenses/restrictions: because the original data is still needed in other projects, it is temporarily unavailable, you can contact the corresponding author’s e-mail address to provide data. Requests to access these datasets should be directed to Junqi Wang, wangjq_68@163.com.

## Footnotes

^1^ http://tissuearray.org/
