# Supplementary material for: Prognostic value of the triglyceride—glucose index in non-muscle-invasive bladder cancer: a retrospective study
Source: Front Nutr. 2024 Dec 16;11:1472104. doi: 10.3389/fnut.2024.1472104 (PMC11682997; doi:10.3389/fnut.2024.1472104)
Supplement: Supplementary file 1 [file Data_Sheet_1.zip › Supplementary Materials.docx]

**Supplementary Materials**

**Tables**

**Supplementary Table 1. Univariable and Multivariable Analysis for Predicting RFS inT1 Patients**

| **Variables** | **Univariate RFS HR (95% CI)** | ***P* Value** | **Multivariate RFS HR (95% CI)** | ***P* Value** |
| --- | --- | --- | --- | --- |
| Age (year) |  | 0.319 |  |  |
| <70 | 1 (reference) |  |  |  |
| ≥70 | 1.452 (0.697 - 3.025) |  |  |  |
| Gender |  | 0.644 |  |  |
| Male | 1 (reference) |  |  |  |
| Female | 1.326 (0.401 - 4.383) |  |  |  |
| BMI |  | 0.807 |  |  |
| <25 | 1 (reference) |  |  |  |
| ≥25 | 0.92(0.469-1.803) |  |  |  |
| TyG |  | 0.221 |  |  |
| <8.61 | 1 (reference) |  |  |  |
| ≥8.61 | 1.581 (0.755 - 3.311) |  |  |  |
| Hb (g/l) |  | 0.196 |  |  |
| <136 | 1 (reference) |  |  |  |
| ≥136 | 0.618 (0.298 - 1.281) |  |  |  |
| Alb (g/l) |  | 0.552 |  |  |
| <43.4 | 1 (reference) |  |  |  |
| ≥43.4 | 0.800 (0.384 - 1.667) |  |  |  |
| Maximum tumour diameter (mm) | 1.030 (1.007 - 1.054) | 0.010 | 1.007 (0.981 - 1.035) | 0.589 |
| Prior recurrence status |  | <0.001 |  | 0.012 |
| Primary | 1 (reference) |  | 1 (reference) |  |
| Recurrent | 4.687 (2.134 - 10.293) |  | 3.047 (1.280 - 7.251) |  |
| Bladder neck invasion |  | 0.033 |  | 0.154 |
| No | 1 (reference) |  | 1 (reference) |  |
| Yes | 2.265 (1.069 - 4.800) |  | 1.835 (0.796 - 4.226) |  |
| Hypertension |  | 0.755 |  |  |
| No | 1 (reference) |  |  |  |
| Yes | 1.133 (0.516 - 2.491) |  |  |  |
| Diabetes |  | 0.991 |  |  |
| No | 1 (reference) |  |  |  |
| Yes | 0.994 (0.345 - 2.860) |  |  |  |
| Smoking status |  | 0.327 |  |  |
| No | 1 (reference) |  |  |  |
| Yes | 1.442 (0.693 - 2.999) |  |  |  |
| Tumor number |  | 0.119 |  |  |
| Single | 1 (reference) |  |  |  |
| Multiple | 1.968 (0.840 - 4.610) |  |  |  |
| Pathology grade |  | 0.306 |  |  |
| Low-grade | 1 (reference) |  |  |  |
| High-grade | 2.836 (0.385 - 20.890) |  |  |  |
| Second TURBT |  | 0.063 |  |  |
| No | 1 (reference) |  |  |  |
| Yes | 0.425 (0.173 - 1.047) |  |  |  |

Abbreviations: BMI, Body mass index; Hb, Hemoglobin; Alb, Albumin; TURBT, transurethral resection of bladder tumours.

**Supplementary Table 2. Univariable and Multivariable Analysis for Predicting PFS in T1 Patients**

| **Variables** | **Univariate RFS HR (95% CI)** | ***P* Value** | **Multivariate RFS HR (95% CI)** | ***P* Value** |
| --- | --- | --- | --- | --- |
| Age (year) |  | 0.242 |  |  |
| <70 | 1 (reference) |  |  |  |
| ≥70 | 1.671 (0.706 - 3.954) |  |  |  |
| Gender |  | 0.841 |  |  |
| Male | 1 (reference) |  |  |  |
| Female | 1.326 (0.401 - 4.383) |  |  |  |
| BMI |  | 0.841 |  |  |
| <25 | 1 (reference) |  |  |  |
| ≥25 | 0.916 (0.389 - 2.158) |  |  |  |
| TyG |  | 0.209 |  |  |
| <8.61 | 1 (reference) |  |  |  |
| ≥8.61 | 1.759 (0.729 - 4.245) |  |  |  |
| Hb (g/l) |  | 0.052 |  |  |
| <136 | 1 (reference) |  |  |  |
| ≥136 | 0.423 (0.178 - 1.006) |  |  |  |
| Alb (g/l) |  | 0.526 |  |  |
| <43.4 | 1 (reference) |  |  |  |
| ≥43.4 | 0.756 (0.318 - 1.796) |  |  |  |
| Maximum tumour diameter (mm) | 1.029 (1.002 - 1.057) | 0.038 | 1.011 (0.982 - 1.041) | 0.448 |
| Prior recurrence status |  | 0.004 |  | 0.076 |
| Primary | 1 (reference) |  | 1 (reference) |  |
| Recurrent | 3.823 (1.518 - 9.630) |  | 2.487 (0.940 - 6.582) |  |
| Bladder neck invasion |  | 0.176 |  |  |
| No | 1 (reference) |  |  |  |
| Yes | 1.873 (0.755 - 4.645) |  |  |  |
| Hypertension |  | 0.996 |  |  |
| No | 1 (reference) |  |  |  |
| Yes | 0.997 (0.387 - 2.573) |  |  |  |
| Diabetes |  | 0.985 |  |  |
| No | 1 (reference) |  |  |  |
| Yes | 0.989 (0.290 - 3.367) |  |  |  |
| Smoking status |  | 0.581 |  |  |
| No | 1 (reference) |  |  |  |
| Yes | 1.327 (0.562 - 3.131) |  |  |  |
| Tumor number |  | 0.166 |  |  |
| Single | 1 (reference) |  |  |  |
| Multiple | 2.035 (0.745 - 5.559) |  |  |  |
| Pathology grade |  | 0.471 |  |  |
| Low-grade | 1 (reference) |  |  |  |
| High-grade | 2.096 (0.280 - 15.668) |  |  |  |
| Second TURBT |  | 0.104 |  |  |
| No | 1 (reference) |  |  |  |
| Yes | 0.405 (0.136 - 1.206) |  |  |  |

Abbreviations: BMI, Body mass index; Hb, Hemoglobin; Alb, Albumin; TURBT, transurethral resection of bladder tumours.

**Figures**

**
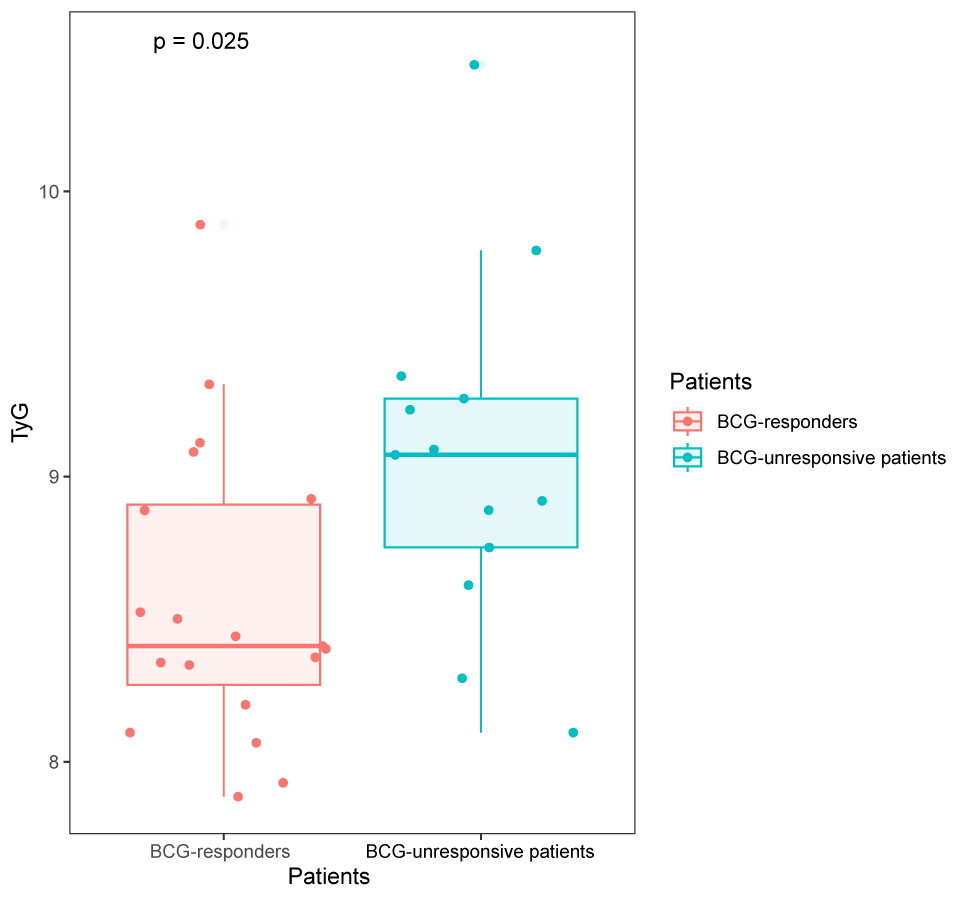
**

**Supplementary Figure 1. Correlations between the TyG Index and the BCG-unresponsive and BCG-responsive patients.**
